# Improving systemic therapy selection for inflammatory skin diseases: A clinical need survey

**DOI:** 10.1016/j.jdin.2024.03.019

**Published:** 2024-04-06

**Authors:** Nicholas D. Brownstone, Aaron S. Farberg, Graham H. Litchman, Ann P. Quick, Jennifer J. Siegel, Lenka V. Hurton, Matthew S. Goldberg, Peter A. Lio

**Affiliations:** a Department of Dermatology, Temple University Hospital, Philadelphia, Pennsylvania; b Baylor Scott & White Health System, Dallas, Texas; c Bare Dermatology, Dallas, Texas; d Vivida Dermatology, Las Vegas, Nevada; e Castle Biosciences, Inc, Friendswood, Texas; f Department of Dermatology, Northwestern University Feinberg School of Medicine, Chicago, Illinois

**Keywords:** atopic dermatitis, biologics, gene expression profile test, inflammatory skin disease, molecular, precision medicine, psoriasis, response to therapy, systemic therapy

## Abstract

**Background:**

Empirical decisions to select therapies for psoriasis (PSO) and atopic dermatitis (AD) can lead to delays in disease control and increased health care costs. However, routine molecular testing for AD and PSO are lacking.

**Objective:**

To examine (1) how clinicians choose systemic therapies for patients with PSO and AD without molecular testing and (2) to determine how often the current approach leads to patients switching medications.

**Methods:**

A 20-question survey designed to assess clinician strategies for systemic treatment of AD and PSO was made available to attendees of a national dermatology conference in 2022.

**Results:**

Clinicians participating in the survey (265/414, 64% response rate) ranked “reported efficacy” as the most important factor governing treatment choice (*P* < .001). However, 62% (165/265) of clinicians estimated that 2 or more systemic medications were typically required to achieve efficacy. Over 90% (239/265) of respondents would or would likely find a molecular test to guide therapeutic selection useful.

**Limitations:**

To facilitate ease of recall, questions focused on systemic therapies as a whole and not individual therapies.

**Conclusion:**

Clinicians want a molecular test to help determine the most efficacious drug for individual patients.

**Capsule Summary**

• Clinicians currently make empirical decisions when selecting systemic therapies for the treatment of moderate-to-severe atopic dermatitis and psoriasis.
• In this study, greater than 90% of surveyed clinicians indicated an interest in a molecular test to supplement their clinical algorithm for more effective systemic therapy selection.

## Introduction

Atopic dermatitis (AD) and psoriasis (PSO) are 2 of the most common inflammatory skin diseases (ISDs), with AD affecting nearly 10% of the pediatric population and nearly 5% of adults and PSO impacting approximately 3% of the US population.^1^, ^2^, ^3^ Both AD and PSO present with a chronic relapsing-remitting course with downstream comorbidities due to systemic inflammation, as well as reduced quality of life from physical, psychosocial, and economic health burdens.^4^, ^5^, ^6^, ^7^

Systemic pharmacologic options for patients with moderate-to-severe AD/PSO include immunosuppressive agents (eg, methotrexate) and targeted immunomodulatory agents, including biologics and targeted synthetic small molecules (tSM).^8^ The therapeutic landscape for moderate-to-severe PSO has greatly expanded over the past 2 decades with 13 United States Food and Drug Administration (FDA)–approved biologic agents, 2 tSM, and several broad immunosuppressive agents. As of 2023, AD has 4 FDA-approved biologic and tSM therapies, and it is anticipated that AD will become more of a challenge than PSO for personalizing treatment.

Despite the improved population-based treatment responses reported with biologic and tSM therapies for both PSO and AD treatment, disease heterogeneity exists along with the complex interplay between a patient’s genomic and environmental/lifestyle factors that result in significant variability in treatment response for any given patient.^9^ This variability in treatment response is why organizations such as the National Psoriasis Foundation and American Academy of Dermatology have recognized the need for personalized medicine within the field.^10^ This clinician-based study examines current approaches to selecting systemic therapies for patients with moderate-to-severe AD and PSO in the absence of routine molecular data, as well as clinician perception of a predictive molecular test.

## Methods

### Study administration

The study was made available via a link to attendees by administrators at the Winter Clinical Dermatology 2022 conference and a total of 265 clinicians completed the questionnaire. Participation was voluntary and not associated with data presentation, and respondents completing the survey received monetary remuneration. The questionnaire was designed to assess clinician strategies for systemic treatment of common ISDs, specifically moderate-to-severe AD and PSO, to better understand differences in treatment practices and risk assessment in patients. Participants were asked about which factors they use to select a systemic therapy for (i) newly diagnosed patients with PSO or for whom topical treatment did not work and (ii) patients changing systemic therapy due to loss of efficacy. Participants also provided their experience with the number of systemic therapies used prior to finding one that is efficacious, as well as their perspectives on the utility of a personalized molecular test to guide systemic therapy selection. Responses to questions for therapy selection factors and molecular test interest were captured using a 5-point Likert Scale to quantify clinician opinions. Clinical and demographic variables that may impact clinician preferences were also collected.

### Statistical analyses

Descriptive statistics were tabulated on all variables. Standard deviation is reported where mean and error are shown. Likert Scale scores were treated as ordinal data and analyzed using the Kruskal-Wallis rank sum test to assess differences between systemic therapy selection factors. Where the overall test was significant, group comparisons were completed using Wilcoxon test between neighboring factors with multiple testing correction via Holm’s method. A *P* < .05 was considered statistically significant.

## Results

### Participant demographics

In total, 265/414 (64%) of attending health care practitioners who were provided the questionnaire link completed the survey and participant demographics are shown in Table I. Respondents were primarily dermatologists (88.7%) with the remaining 11.3% of participants’ specialties including dermatopathologist, dermatologist/dermatopathologist, nurse practitioner/physician assistant, Mohs, or other specialists. Clinicians in practice from 1 to 10 years (33.2%) and residents/fellows (27.6%) were the prevalent experience levels of respondents, and clinicians in practice 11 to 20, 21 to 30, and >30 years were represented at 15.9%, 12.5%, and 10.9% of respondents, respectively. Respondents were primarily in a group practice (38.5%), solo practice (15.1%), or multispecialty groups (10.6%). Providers at academic locations represented 35.4% of respondents. Clinicians reported monthly treatment volume of patients with moderate-to-severe AD/PSO at 1 to 10 for 33.2% of respondents, 11 to 24 for 37.7% of respondents, with 27.2% of clinicians seeing a higher volume of patients (25-49 and ≥ 50 patients/month). Regarding monthly prescription volume of biologics for AD/PSO, 47.6% of clinicians prescribed 1 to 10, 36.2% of clinicians prescribed 11 to 24, while 8.3% and 4.5% of clinicians prescribed 25 to 49 and ≥ 50, respectively.

**Table I:** Demographics of surveyed clinicians (*N* = 265)

Demographic variable	No. of respondents (%)
Primary specialty	
Dermatologist	235 (88.7)
Dermatopathologist or dermatologist/dermatopathologist	3 (1.1)
Nurse practitioner/physician assistant	19 (7.2)
Mohs fellow	1 (0.4)
Other specialist	7 (2.6)
Years in practice	
Resident or fellow	73 (27.6)
1-10	88 (33.2)
11-20	42 (15.9)
21-30	33 (12.5)
>30	29 (10.9)
Practice type	
Academic/university	94 (35.4)
Group practice	102 (38.5)
Multispecialty group	28 (10.6)
Solo practice	40 (15.1)
Retired	1 (0.4)
Patients with moderate-to-severe AD/PSO per month (No.)	
0	5 (1.9)
1-10	88 (33.2)
11-24	100 (37.7)
25-49	41 (15.5)
≥50	31 (11.7)
Biologics prescribed for AD/PSO per month (No.)	
0	9 (3.4)
1-10	126 (47.6)
11-24	96 (36.2)
25-49	22 (8.3)
≥50	12 (4.5)

*AD*, Atopic dermatitis; *PSO*, psoriasis.

### Factors currently guiding initial systemic therapy selection

**Fig 1:**
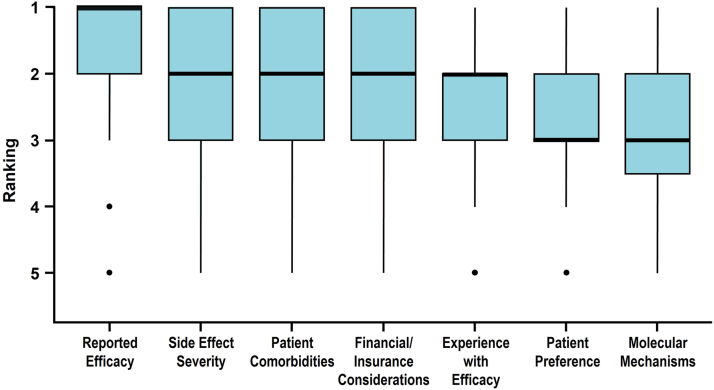
Psoriasis. Factors clinicians consider when choosing an initial systemic therapy for patients with psoriasis (*N* = 265). Each therapy selection factor was rated on a 5-point Likert scale from most important (1) to least important (5) with compiled data shown as boxplots. The number of responses collected per attribute ranged from 255 to 262. The *horizontal bar* indicates the median value, *box ends* demarcate the first and third quartiles, *whiskers* show the range of observations within 1.5 times the interquartile range below the first quartile and above the third quartile, and *circles* represent outliers.

Clinicians force ranked 7 factors they may consider when selecting the first systemic therapy for patients diagnosed with PSO. Respondents’ ratings of therapy factors showed median rank scores (mean, standard deviation also given) of: 1 (1.67, 1.02) for “reported efficacy,” 2 (2.10, 1.06) for “side effect severity,” 2 (2.18, 1.03) for “patient comorbidities,” 2 (2.32, 1.18) for “financial/insurance considerations,” 2 (2.43, 1.18) for “experience with efficacy,” 3 (2.65, 1.14) for “patient preference,” and 3 (2.75, 1.16) for “molecular mechanisms” (Fig 1). Clinicians do not consider all factors equally (ꭓ^2^[6] = 178.6, *P* < .001). “Reported efficacy” was the factor most often ranked with highest importance and was significantly different from the next highest-ranking factor of “side effect severity” (*P* < .001). “Patient preference” and “molecular mechanism” were ranked with the lowest importance (*P* = .019, “experience with efficacy” vs “patient preference”; *P* = .4, “patient preference” vs “molecular mechanisms”).

### Second line systemic therapy due to loss of efficacy from initial systemic therapy

**Fig 2:**
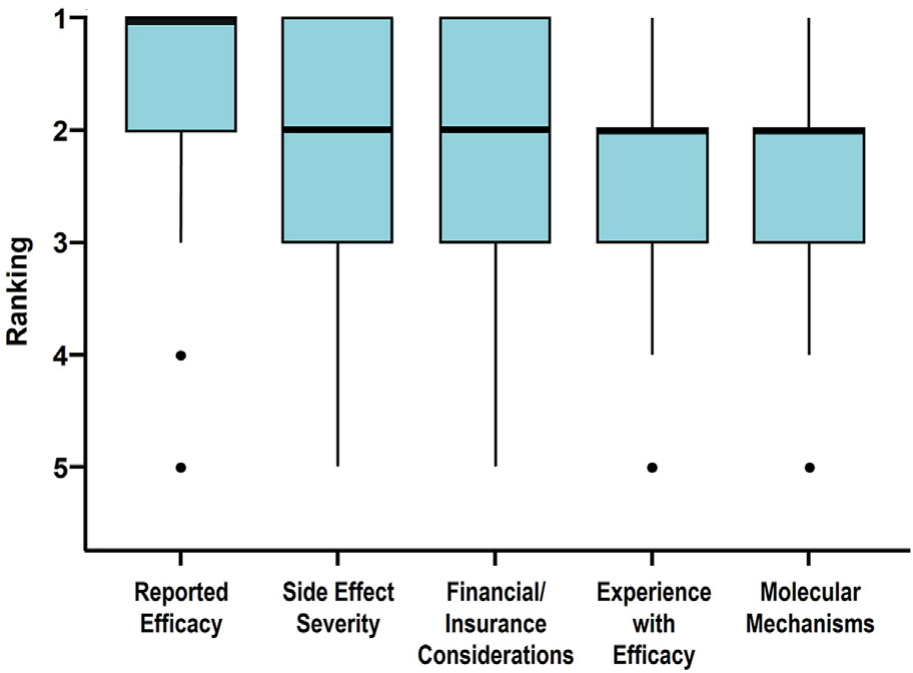
Psoriasis and atopic dermatitis. Factors clinicians consider when choosing a second line systemic therapy due to loss of efficacy from the first therapy in patients with atopic dermatitis or psoriasis (*N* = 265). Each therapy selection factor was graded on a 5-point Likert scale from most important (1) to least important (5) with compiled data shown as boxplots. The number of responses collected per attribute ranged from 262 to 265. The *horizontal bar* indicates the median value, *box ends* demarcate the first and third quartiles, *whiskers* show the range of observations within 1.5 times the interquartile range below the first quartile and above the third quartile, and *circles* represent outliers.

Clinicians force ranked 5 factors they may consider when switching AD/PSO patients to a different systemic therapy due to loss of efficacy. Respondents’ rankings of second line systemic therapy factors showed a median ranking (mean, standard deviation) of: 1 (1.69, 1.00) for “reported efficacy,” 2 (2.09, 1.10) for “side effect severity,” 2 (2.21, 1.09) for “financial/insurance considerations,” 2 (2.32, 1.11) for “experience with efficacy,” and 2 (2.50, 1.23) for “molecular mechanisms” (Fig 2). Clinicians do not consider all factors equally (ꭓ^2^[4] = 89.147, *P* < .001). When switching treatments clinicians indicated that “reported efficacy” was still considered the most important factor (*P* < .001, “reported efficacy” vs “side effect severity”) in the practitioner’s decision-making process, while other factors were not differently weighted in the decision-making (*P* > .05).

### Reasons for switching systemic therapy

Clinicians were asked to select the most common reason a patient discontinues systemic therapy. Clinicians’ top factor selection was “no symptom improvement” (37%), followed closely by “insurance declined” (32%), “patient financial burden” (15%), “side effects” (13%), and “other” (3%) (Table II).

**Table II:** The most common factors clinicians identified for patients with moderate-to-severe atopic dermatitis or psoriasis in discontinuing systemic therapy (*N* = 265)∗

Patient treatment challenge group	No. of respondents (%)
No symptom improvement	100 (37.7)
Insurance declined	84 (31.7)
Patient financial burden	39 (14.7)
Side effects	33 (12.5)
Other	9 (3.4)

∗ Respondents were asked to select their top reason (a single response permitted).

### Number of systemic therapies needed to find an effective treatment

**Fig 3:**
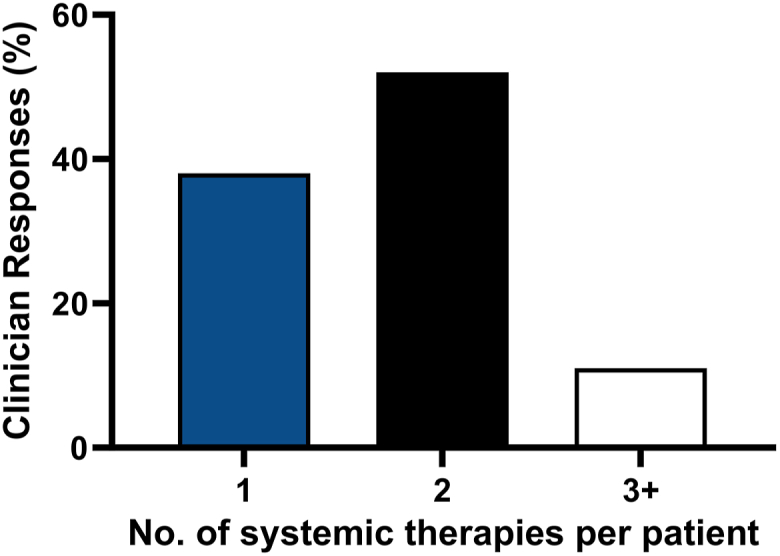
Psoriasis. Clinician estimates of the average number of systemic therapies for their patients with psoriasis to identify an efficacious drug (*N* = 265). Respondents were asked to identify the average number of systemic therapies attempted before finding one that is efficacious for their patients with psoriasis on systemic therapy. Selections for the average number of systemic therapies per patient were 1 (*blue bar*), 2 (*black bar*), or ≥3 (*white bar*).

Clinicians estimated the average number of systemic therapies their patients with PSO received before finding an effective medication. Thirty-eight percent (*n* = 100) of clinicians estimated that on average their patients achieved efficacy with the first systemic therapy. However, 52% (*n* = 137) estimated that on average it took 2 systemic therapies and 11% (*n* = 28) of respondents attempted ≥3 systemic therapies before attaining therapeutic efficacy (Fig 3).

### Clinician interest in molecular testing

**Fig 4:**
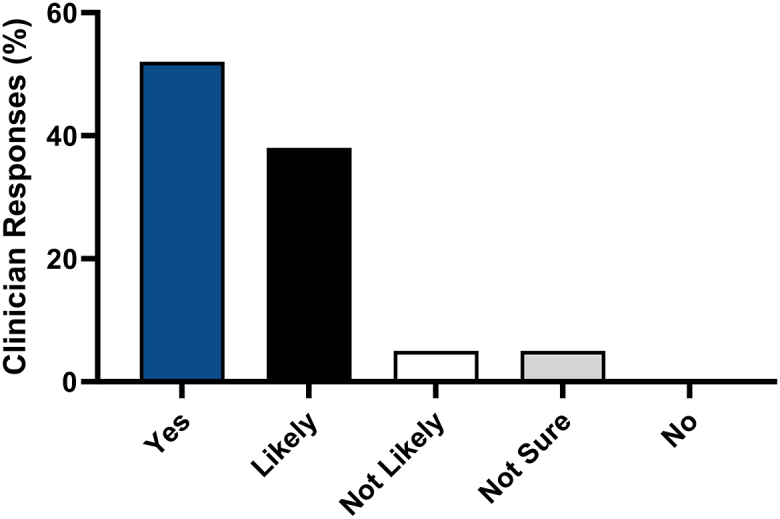
Psoriasis and atopic dermatitis. Clinician interest levels in using a predictive biomarker test providing guidance on therapy selection for patients with atopic dermatitis or psoriasis (*N* = 265). Respondents were asked to rate how useful they would find a personalized molecular test informing on therapy selection for patients with moderate-to-severe atopic dermatitis or psoriasis. Responses were rated on a 5-point Likert scale from useful (“yes”), “likely” useful, “not likely” useful, “not sure”, to not useful (“no”).

Participants’ opinions were assessed as to whether they would find an accessible personalized molecular test that guided systemic therapy selection for patients with moderate-to-severe AD/PSO useful. Fifty-two percent (*n* = 137) of respondents indicated “yes” and 38% (*n* = 102) replied “likely;” whereas 5% (*n* = 14) of respondents indicated “not likely,” 5% (*n* = 12) were “not sure,” and 0 “no” responses (Fig 4).

**Fig 5:**
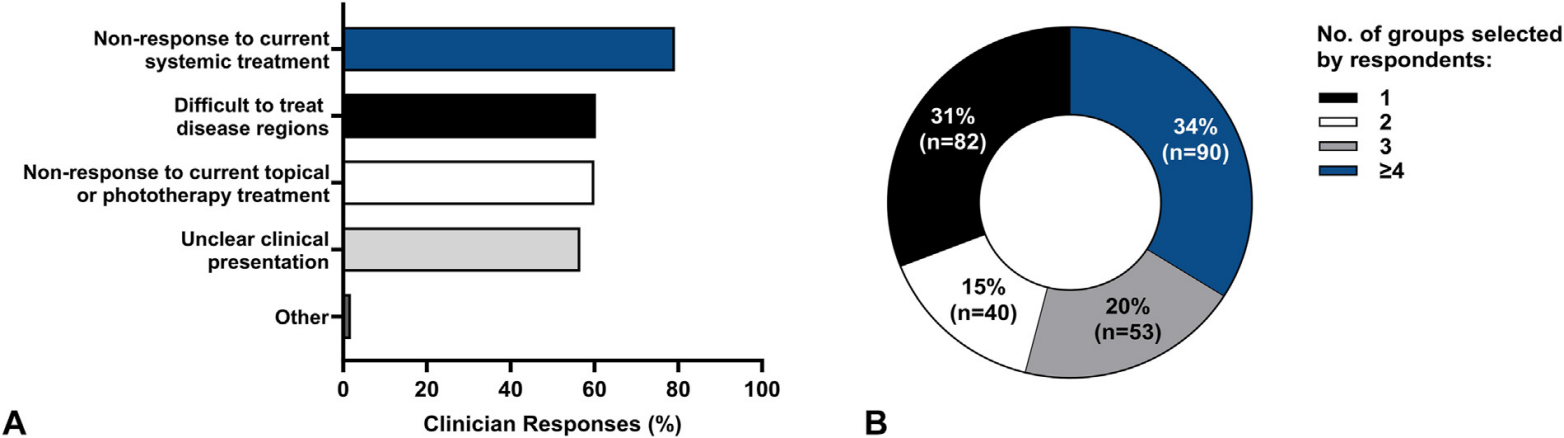
Psoriasis and atopic dermatitis. Respondent selection of patient groups with psoriasis and atopic dermatitis with unmet therapeutic needs for whom clinicians would be interested in using a personalized molecular test to guide systemic therapy selection. **A,** Respondents (*N* = 265) were asked to select all applicable moderate-to-severe patient groups with atopic dermatitis or psoriasis for which the clinicians would prefer to use a molecular test to guide their systemic therapy selection. Difficult to treat disease regions included nail/scalp/genital involvement or body surface area >10%. **B,** The patient group selections from (**A**) were summed per respondent and presented as parts of a whole.

If such a predictive molecular test were available, respondents were asked to select potential patient groups with AD/PSO (multiple selections permitted) for which the clinician would prefer to use the molecular test. All 265 respondents selected at least 1 patient group. Respondents indicated that they would use a predictive molecular test to guide systemic therapy selection for patients with “nonresponse to current systemic treatment” (79.2%, *n* = 210), “difficult to treat disease regions” (60.4%, *n* = 160), “nonresponse to current topical or phototherapy treatment” (60%, *n* = 158), “unclear clinical presentation” (56.6%, *n* = 150), and “other” (1.9%, *n* = 5) (Fig 5, *A*). Since clinicians could select multiple patient groups, 31% (*n* = 82) of respondents chose 1 patient group and 15% (*n* = 40) selected 2 groups. Whereas 20% (*n* = 53) and 34% (*n* = 90) of respondents selected a combination of 3 or ≥4 patient groups, respectively (Fig 5, *B*). With respect to the potential for broad test utilization, 69% of respondents selected 2 or more groups of patients who could benefit from a predictive molecular test.

## Discussion

Biologic and tSM therapies have been developed for use in AD and PSO to improve treatment responsiveness and/or reduce adverse events compared to immunosuppressive therapies. Targets were chosen based upon a mechanistic understanding of the underlying inflammatory processes. While these aims at improving satisfactory therapeutic response with similar or reduced adverse events on a population-based level have been achieved, about 22% of patients with PSO switch systemic therapies, demonstrating that individual patients with PSO continue to experience high variability in adequate therapy responsiveness (source: IQVIA medical and prescription claims data 2017-2021, unpublished data). With the recent FDA approval of 3 alternatives to dupilumab, similar switching rates are anticipated for patients with AD due to high heterogeneity of clinical presentation of AD and response to therapy compared to PSO. With this increasing complexity and paradigm shift toward precision medicine, it is becoming more important, yet more difficult, for practitioners to make informed decisions about each individual patient’s therapeutic plan. This clinician-based survey assessed this topic with respect to moderate-to-severe AD and PSO to gain a better understanding of the factors currently guiding systemic therapy selection. Whether selecting a first systemic therapy or switching to the next systemic therapy due to loss of efficacy, respondents indicated “reported efficacy” as the most important factor while “molecular mechanism” was one of the factors considered the least important in the current decision-making process, which is not surprising due to the paucity of clinically available molecular testing for ISD. The continued clinician reliance on “reported efficacy” when switching therapy alludes to the importance of evidence-based decision-making in current practice and highlights the need for additional objective molecular information.

Mechanism of action is the foundation upon which these biologics and tSM are developed and as such they possess targeted activity, yet there is a disconnect with the trial-and-error approach currently employed to select systemic therapies. Despite high reported efficacies among FDA-approved therapies for moderate-to-severe PSO and AD, “no symptom improvement” was the top reason reported for patient discontinuation of systemic medications for AD/PSO in our study, indicating that not all medications are efficacious for all patients. In this study, 62% of clinicians surveyed estimated that, on average, 2 or more systemic therapies were needed to find one that was efficacious. These results suggest that a subset of patients do not initially receive the optimal systemic medication for their AD/PSO leading to patients cycling through drugs. For PSO, switching or discontinuing treatment is a common occurrence in the real-world setting with a major factor for patients discontinuing biologic treatments reported as lack of efficacy and one where a trial-and-error approach is used for treatment selection.^5^^,^^6^^,^^11^^,^^12^ A consequential requisite of biologics regimens that patients encounter when starting or switching biologics is having to undergo an initial higher loading dose to reach therapeutic response prior to adopting a lower maintenance dose. Studies of real-world treatment patterns and health care costs for such patient groups report that patients with PSO switching biologics incur higher health care costs, largely by increased prescription costs as well as medical costs.^6^^,^^7^ In AD, FDA-approved biologic and tSM options have expanded beyond dupilumab only recently and now include abrocitinib, upadacitinib, and tralokinumab with multiple new compounds in development and clinical trials. It would be reasonable to expect similar challenges to those that exist in PSO. Despite good clinical trial data to guide therapy selection, disparity in drug efficacy exists in real-world practice^13^ reflecting the presence of disease/patient/genomic heterogeneity underpinning the current use of a trial-and-error approach. This trial-and-error approach to therapy selection may lead to delays in appropriate treatment, decreased quality of life, and increased cost to health care systems.^14^

In contrast to the generalizable knowledge from population-based studies relied upon by evidence-based medicine, precision medicine aims to connect the intricacies of clinical and molecular characteristics, subpopulations, heterogeneities, and treatment response to an individual patient with the expectation that personalized medicine decisions will improve and facilitate personalized medicine. Many medical subspecialties have incorporated precision medicine into clinical practice and inclusion of multigene testing assays are the subject of favorable systematic review and meta-analyses, expert consensus statements and guidelines.^15^, ^16^, ^17^ At present, noninvasive molecular tests to guide therapeutic selection are not routinely used in practice for PSO/AD and greater than 90% of clinicians would find utility in having a molecular test to help determine the most efficacious drug for individual patients (Fig 4). While nearly 80% of clinicians indicated interest in using such a test for patients not responding to current systemic treatment, over 69% of respondents selected multiple patient groups for which they could envision using the test (Fig 5). This highlights the clinical need for more personalized care that could encompass many areas of ISD and from which patients could benefit. Furthermore, information garnered from this survey may be used to inform future and ongoing research and clinical studies aiming to develop a tool to guide personalized therapeutic selection.

Some limitations to the study exist, such as recall bias, interpretation of questions, and limited answers due to closed-ended questions. To avoid problems of data recall, thus minimizing recall bias, questions were more generalized and systemic therapies were not divided further into drug classes. With the administration of this survey at a national dermatology conference, it is recognized that the survey drew from this attendee pool, of which 15% of practitioners were solo practice and 49% were single- and multispecialty practice groups. By comparison, the Physician Compare Database reported 16% solo dermatologists, while the Centers for Medicare and Medicaid Services databases reported 20% sole practitioners and 58% single- and multispecialty group dermatologists.^18^^,^^19^ Overall, the survey includes a diverse group of practicing clinicians from varied dermatologic practice settings supporting a representative response. Another limitation was the PSO-weighted switching data; however, at the time of the questionnaire development, dupilumab was the only targeted systemic drug available for use in AD. With nearly 2 years of commercial availability for abrocitinib and upadacitinib, future studies are needed to investigate evolving clinician perspectives on selection of targeted therapies for AD.

## Conclusion

This study contributes to the clinician perspective on identifying challenges in optimal therapeutic management for patients with moderate-to-severe PSO and AD amid the burgeoning number of biologics and targeted small molecule therapies. The findings indicate that in the absence of a molecular test to help guide systemic therapy selection for patients with PSO and AD, clinicians currently must make empirical decisions based on limited evidenced-based information, drug formulary/insurance restrictions, personal experience, and available population-based evidence, which can lead to delays in disease control and increased cost to the health care system. Moreover, clinicians responded that they would find utility in a molecular test to identify the optimal therapy for PSO and AD, highlighting the clinical need for such a test as the number of available treatments continues to grow.

## Conflicts of interest

Dr Brownstone received a stipend paid for by Castle Biosciences, Inc. Dr Farberg is a consultant for Castle Biosciences, Inc and on the advisory board for Amgen, Boehringer Ingelheim, Eli Lilly, Galderma, Incyte, Janssen, Novartis, Ortho Dermatologics, Pfizer, and Sun Pharma. Drs Quick, Siegel, Hurton, and Goldberg are employees and option and stockholders for Castle Biosciences, Inc. Dr Lio reports research grants/funding from AbbVie, AOBiome, Eczema Foundation, National Eczema Association; is on the speaker's bureau for AbbVie, Eli Lilly, Galderma, Hyphens Pharma, Incyte, La Roche-Posay/L’Oreal, MyOR Diagnostics, ParentMD, Pfizer, Pierre-Fabre Dermatologie, and Regeneron/Sanofi Genzyme; reports consulting/advisory boards for AbbVie, Almirall, Amyris, Arbonne, ASLAN, Bodewell, Boston Skin Science, Bristol-Myers Squibb, Burt’s Bees, Castle Biosciences, Codex Labs, Concerto Biosci, Dermavant, Dermira, DermVeda, Eli Lilly, Galderma, IntraDerm, Janssen, Johnson & Johnson, Kaleido Biosci, Kimberly Clark, LEO Pharma, Lipidor, L’Oreal, Menlo Therapeutics, Merck, Micreos, MyOR Diagnostics, Regeneron/Sanofi Genzyme, Sibel Health, Skinfix, Sonica, Theraplex, UCB, Unilever, Verrica, and Yobee Care; reports stock options with LearnSkin/Learn Health, Medable, Micreos, Modernizing Medicine, and Yobee Care. In addition, Dr Lio has a patent pending for a Theraplex product with royalties paid and is a Board member and Scientific Advisory Committee Member of the National Eczema Association. Author Litchman has no conflicts of interest to declare.
